# Effects of Chemical Insecticide Imidacloprid on the Release of C_6_ Green Leaf Volatiles in Tea Plants (*Camellia sinensis*)

**DOI:** 10.1038/s41598-018-36556-0

**Published:** 2019-01-24

**Authors:** Qiying Zhou, Xi Cheng, Shuangshuang Wang, Shengrui Liu, Chaoling Wei

**Affiliations:** 1 State Key Laboratory of Tea Plant Biology and Utilization, Anhui Agricultural University, 130 Changjiang West Road, Hefei, Anhui 230036 China; 2 Henan Key Laboratory of Tea Plant Biology, Xinyang Normal University, 237 Nanhu Road, Xinyang, 464000 Henan China; 3 Institute for Conservation and Utilization of Agro-Bioresources in Dabie Mountains, Xinyang Normal University, 237 Nanhu Road, Xinyang, 464000 Henan China

## Abstract

Chemical insecticides are widely used for pest control worldwide. However, the impact of insecticides on indirect plant defense is seldom reported. Here, using tea plants and the pesticide imidacloprid, effects of chemical insecticides on C_6_-green leaf volatiles (GLVs) anabolism and release were investigated first time. Compared with the non-treated control plants, the treatment of imidacloprid resulted in the lower release amount of key GLVs: *(Z)*-3-hexenal, *n*-hexenal, *(Z)*-3-hexene-1-ol and *(Z)*-3-Hexenyl acetate. The qPCR analysis revealed a slight higher transcript level of the *CsLOX3* gene but a significantly lower transcript level of *CsHPL* gene. Our results suggest that imidacloprid treatment can have a negative effect on the emission of GLVs due to suppressing the critical GLVs synthesis-related gene, consequently affecting plant indirect defense.

## Introduction

In response to insect herbivore and pathogen attacks, tea plants can exhibit direct and indirect defenses. Direct defenses employs structural or toxic components to against the aggressor. By contrast, indirect defenses utilizes volatiles to attract natural enemies of the attackers^1–4^. Green leaf volatiles (GLVs) including six-carbon (C_6_) aldehydes, alcohols, and their esters, are important components of plant indirect defenses^5–7^.

GLVs are formed by a two-step reaction catalyzed by lipoxygenase (LOX) and fatty acid 13-hydroperoxide lyase (13HPL), which use linolenic or linoleic acids as substrate. Linolenic acid 13-hydroperoxide (13HPOT) is first synthesized through dioxygen reaction of linolenic acid at position 13, which is catalyzed by LOX. Then, 13HPOT is transformed to two carbonyl compounds by 13HPL. *(Z)*-3-Hexenal, one of the carbonyl compounds of HPL, is reduced to form *(Z)*-3-hexenol. Then, part of *(Z)*-3-hexenol is converted to *(Z)*-3-Hexenyl acetate. When linoleic acid is used as the starting substrate, *n*-hexanal is catalyzed by LOX and 13HPL^8–10^.

Tea is made from leaves of the plant *Camellia sinensis* (L.) O. Kuntze, and is one of the most well-known non-alcohol beverages worldwide. However, tea plants are always attacked by various insect pests, such as tea tussock moth (*Euproctis pseudoconspersa*), tea geometrid (*Ectropis oblique*), tea green leafhopper (*Empoasca flavescens*), etc^11^. In order to kill pests and inhibit diseases, insecticides were always abused in tea plantation management^12^. Considering the effect of insecticide on human health and environmental pollution, however, the impact of insecticides on plant indirect defenses is seldom reported. In this study, the effects of chemical insecticide imidacloprid on the release and anabolism of C_6_ GLVs in tea plants were investigated.

## Results and Discussion

### The effects of imidacloprid on GLVs emission

**Figure 1:**
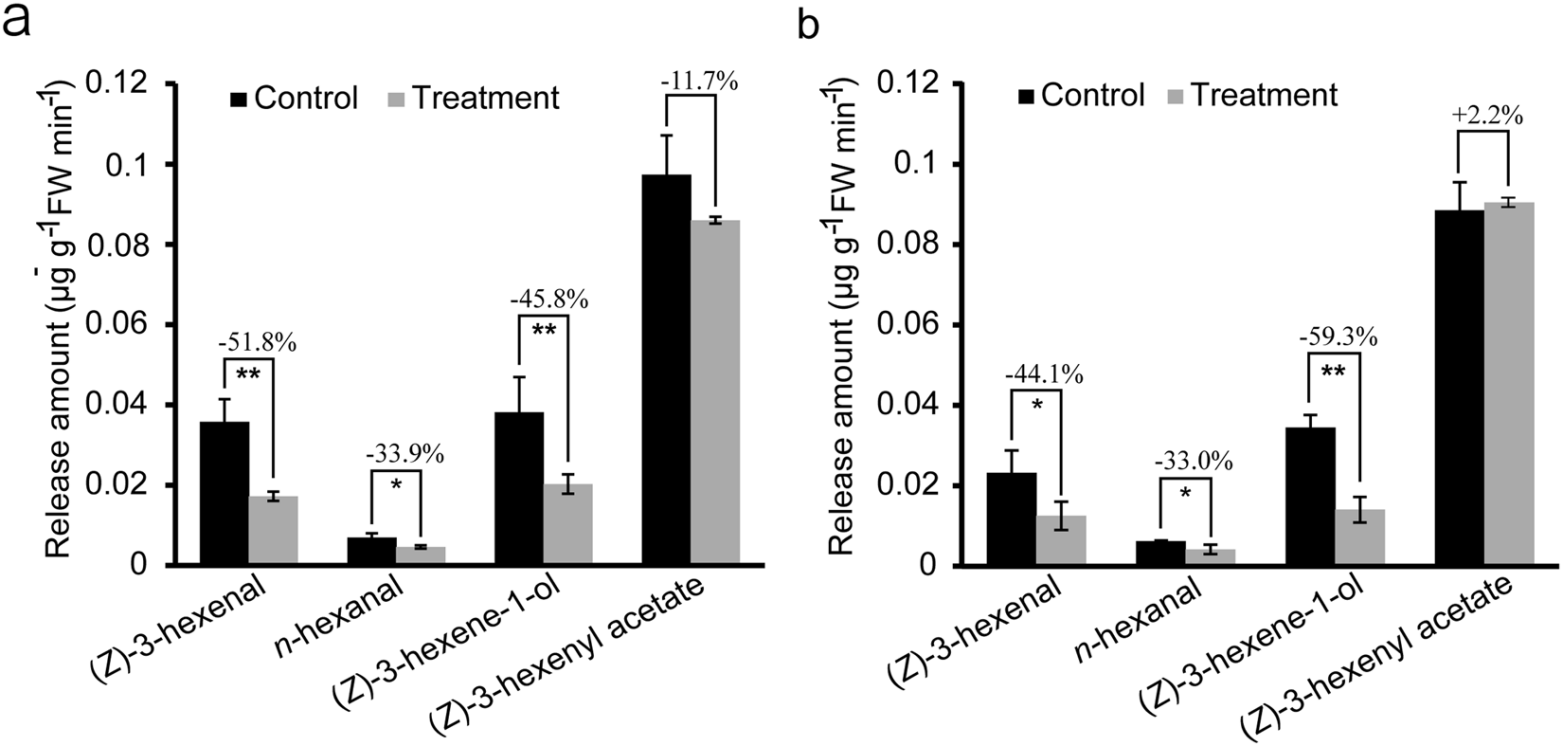
Emission of C_6_ green leaf volatiles after the imidacloprid treatment in tea plants. Values are means ± SD of three independent experiments. Ratios of the reduced release amount of the C_6_ green leaf volatiles from the imidacloprid treated tea plants to that from the control plants were denoted on the lines between the control and the treatment bars. Significant differences are indicated by **P < 0.01 and *P < 0.05; Student’s *t*-test. (**a,b**) denotes the release amounts of four C_6_ green leaf volatiles *(Z)*-3-Hexenal, *n*-hexenal, *(Z)*-3-Hexene-1-ol and *(Z)*-3-Hexenyl acetate in tea plants in the pots and field experiments, respectively.

Imidacloprid treatment had a negative effect on the emission of GLVs in tea plants. In the pot experiment, imidacloprid treated tea plants had reduced emission of the C_6_ green leaf volatiles (GLVs) *(Z)*-3-hexenal, *n*-hexenal, *(Z)*-3-hexene-1-ol and *(Z)*-3-Hexenyl acetate by 51.0%, 33.9%, 45.8% and 11.7%, respectively (Fig. 1a). In the field experiment, the average release amount of *(Z)*-3-Hexenyl acetate was 2.2% higher with the imidacloprid treatment, while the emission of *(Z)*-3-hexenal, *n*-hexenal, *(Z)*-3-hexene-1-ol and *(Z)*-3-Hexenyl acetate was reduced by 44.1%, 33.0% and 59.3%, respectively (Fig. 1b). Inconsistent effects of imidacloprid on *(Z)*-3-Hexenyl acetate emission between the pots and the field experiments could be caused by several reasons: different growth and developmental status of the tea plants, error produced in the GLVs collection processes, and different ecosystems. Nevertheless, research has demonstrated negative effects of pesticides (including insecticides, herbicides, and fungicides) on plant direct defense reaction. The activity of polyphenol oxidase was suppressed and disease resistance ability declined in tobacco, after the treatment with fungicide carbendazim^13^. Agrawal *et al*. reported that defensive chemical ellagitannin content was reduced in esfenvalerate treated *Oenothera biennis*^14^. Here, by measuring the release amount of GLVs, effects of chemical insecticides on indirect plant defense was investigated in tea plants.

GLVs are a class of bioactive oxylipins derived from the octadecanoid pathway, and serve as an important way in plant defense^1,6,15^. In tea plant, research showed an attractant function of GLVs in host location of pests. The mixture of eight compounds including *(E)*-2-hexenal, *(Z)*-3-hexen-1-ol, *(Z)*-3-Hexenyl acetate, 2-penten-1-ol, *(E)*-2-pentenal, pentanol, hexanol and 1-enten-3-ol was more effective in attracting tea green leafhopper (*Empoaca vitis*) than the mixture of linalool, *(Z)*-3-hexen-1-ol and *(E)*-2-hexenal^16^. More recent studies found a higher level of *(Z)*-3-Hexenyl acetate was emitted from the tea varieties that are susceptible to the tea green leafhopper than those tolerant to the insects, which suggested that *(Z)*-3-Hexenyl acetate may function in the attractiveness of tea green leafhopper^17,18^. Furthermore, the addition of *(Z)*-3-Hexenyl acetate to the ternary mixture of *(Z)*-3-hexenal, *(Z)*-3-Hexenyl hexanoate and benzyl alcohol could enhance their effectiveness to attract tea geometrid^19^. Similarly, a susceptible wheat variety released a higher amount of *(Z)*-3-Hexenyl acetate than a resistant variety, thus making them attract more wheat stem sawfly, *Cephus cinctus* Norton^20^. The attractiveness of GLVs *(Z)*-3-Hexenyl acetate to the female click beetle pest *Agriotes brevis* also seemed to be more important than other components in the mixed chemical attractant^21^. Stevens *et al*. (2017) found that *Parastethorus nigripes* and *Stethorus vagans* in Coccinellidaes are attracted to *(Z)*-3-Hexenyl acetate in citrus trial^22^. Together, these studies suggested that *(Z)*-3-Hexenyl acetate may be the most important constituent of GLVs to attract pests for host location. Our results showed that the emission amounts of *(Z)*-3-hexenal, *n*-hexenal and *(Z)*-3-hexen-1-ol were significantly decreased in the imidacloprid treated tea plants compared with that in the control plants, while that of *(Z)*-3-Hexenyl acetate showed little change (Fig. 1). Although studies have demonstrated an attractant function of *(Z)*-3-Hexenyl acetate, whether attractiveness of the GLVs to the pests was affected in tea plants after the imidacloprid treatment was uncertain, considering the attractiveness of GLVs to pests was always studied by a volatile mixture. This indeed an interesting question for further investigation.

GLVs are also known to attract the natural enemies of herbivores^1,6,15,23^. Emitted GLVs were found to attract the enemies of pests commonly found in tea plantation. *(Z)*-3-hexenol can enhance both direct and indirect plant defenses against tea geometrid (*Ectropis oblique*). Tea plants treated with *(Z)*-3-hexenol were found to induce the resistance to tea geometrid and emit more GLVs, and thus attracting more *Apanteles* sp., an important parasitic natural enemy of the tea geometrid^24,25^. It is also reported that the GLVs constituents *(Z)*-3-hexenal, *(Z)*-3-hexen-1-ol and *(Z)*-3-Hexenyl acetate were more effective to attract female *Apanteles* sp. (Hymenoptera: Braconidae) than that to the male *Apanteles* sp., and induced higher electroantennogram (EAG) response in female *Apanteles* sp. than in male *Apanteles* sp^26^. Salicylhydroxamic acid treatment reduced the *LOX* and *HPL* gene expression, thus suppressed GLVs release and plants ability to attract the parasitoid wasp *Apanteles* sp. of tea geometrid^27^. *Xysticus ephippiatus* Simon, a predator of tea geometrid larvae, prefers the tea geometrid damaged tea leaves than that in undamaged ones which emitted little GLVs, indicating GLVs are important in inducing prey for *Xysticus ephippiatus* Simon^28^. Attractiveness of GLVs to the natural enemies (*Chrysopa sinica* and *Aphidius* sp.) has also been demonstrated for tea aphid, *Toxoptera aurantii* Boyer (Hemiptera: Aphididae), another pest, which may cause serious damage of a tea farm. *Z*-3-Hexen-1-ol is the main component of the volatiles generated from the tea aphid-damaged tea shoots, which can attract the natural enemies, and elicited a stronger EAG response of *Chrysopa sinica* and *Aphidius* sp. in a dose-dependent manner^29,30^. All these findings clearly demonstrated that the GLVs did play an important role in the indirect defense of tea plants by attracting the natural enemies of the pests.

So, we can see that the GLVs function as a double-edged sword in plants. The GLVs on one side have a function to attract pests, and can induce plant defense on the other side. Although the GLVs (especially *(Z)*-3-Hexenyl acetate) showed a role in pests attraction, many studies have demonstrated that the composition and proportion of the GLVs attractant were both important^31^. Previous reports have revealed dose-dependent attractiveness of GLVs to pests^30,32,33^, as well as to the natural enemies of pests^15,24,27,30^. For instance, silkworms can secrete a protein which inhibits the biosynthesis of GLVs in plants, leading to the suppressed emission of GLVs and fewer eggs laid by the parasitoid fly of silkworms^34^. In the current study, the release amount of the GLVs in the imidacloprid treated plants was much lower than that in the water treated control plants in both the pots and the field experiments, suggesting a negative effect of imidacloprid on GLVs release. Such reduction in GLV emission thus discounted defense of tea plants to the pests.

### The expression level of GLVs-biosynthesis genes

**Figure 2:**
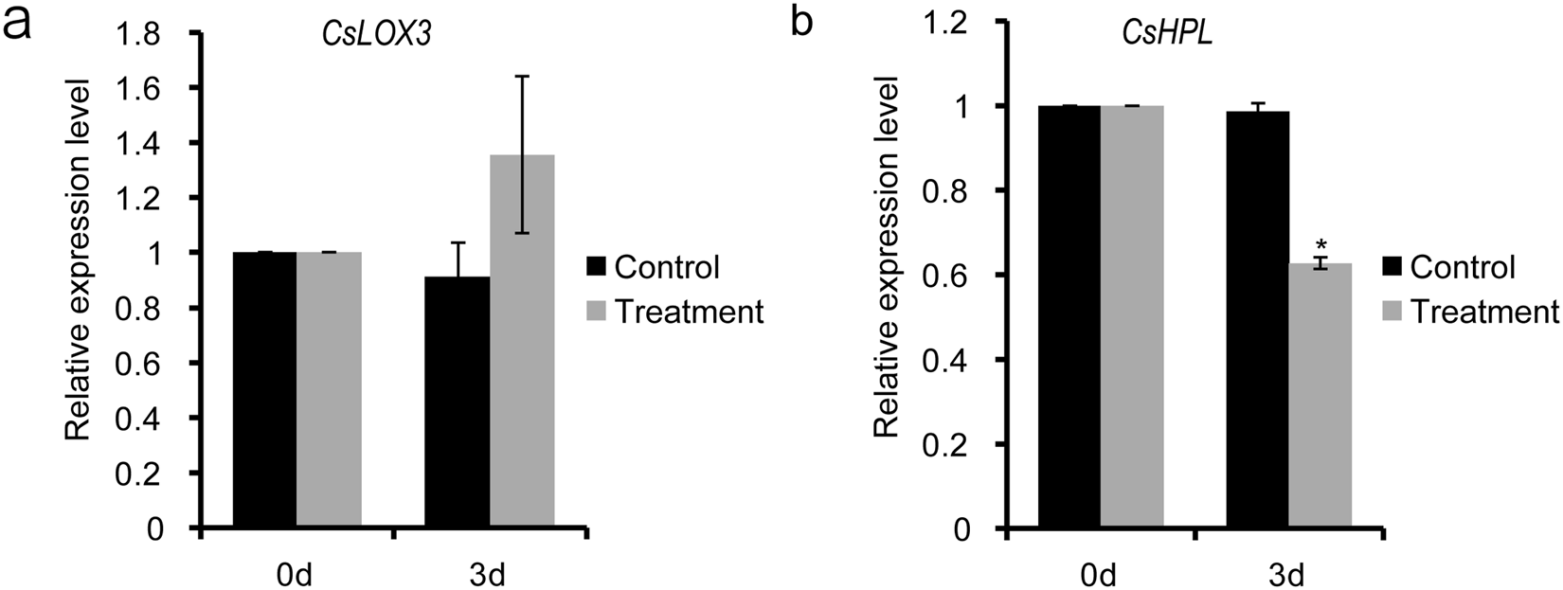
The expression levels of *CsLOX3* and *CsHPL* genes after the imidacloprid treatment in tea plants. Data are means ± SD of three independent experiments. Water treated tea plants are used as controls. Significant differences are indicated by *P < 0.05; Student’s *t*-test. (**a,b**) respectively denotes the expression level of *CsLOX3* and *CsHPL* genes after the imidacloprid treatment in tea plants in the pots experiment. 0d, Leaves collected from tea plants without imidacloprid treatment; 3d, Leaves collected from tea plants on the third day after the imidacloprid or water treatment.

To see whether gene expression for GLVs generation was affected after imidacloprid treatment, qPCR analysis of *CsLOX* (lipoxygenase) and *CsHPL* (hydroperoxide lyase) genes was investigated. In tea plant, *CsLOX* can be classified as 9-*LOX*, 13-*LOX* and 9/13-*LOX*, according to the substrate specificity. Similarly, *CsHPL* can be classified as 9-*HPL*, *13-HPL* and 9/13-*HPL*^9,35^. *CsHPL* (Genbank: HM 440156), with 13-HPL activity exists as a single copy in tea genome. Yet, it plays an important function in *(Z)*-3-hexenal formation and the defense against *Ectropis obliqua* feeding^9^. For *CsLOX* gene, recent report demonstrated that there are 11 *CsLOX*s gene members in tea plant. *CsLOX3* gene (Genbank: HM440161 and FJ794853.1), classified as 13-LOX can catalyze the transformation of α-linolenic acid to 13-HPOT was analyzed in this study^35^. Results showed that the expression level of *CsLOX3* gene was slight higher in the imidacloprid treated tea plants compared with that in the control plants (Fig. 2a). However, the expression level of *CsHPL* gene, which catalyze the C_6_ aldehydes generation, was much lower in the imidacloprid treated tea plants than that in the control plants (Fig. 2b). The results suggested that insecticide imidacloprid could reduce GLVs emission via the suppression of *CsHPL* in tea plants.

In plant, *LOX* gene is not only responsible for GLVs generation, but also involved in jasmonate (JA) synthesis, which can regulate many biological processes, including plant development, response to environmental stress, hypersensitive reaction, wounding and defense reaction^1,36^. The slight higher expression level of the *CsLOX3* gene in the imidacloprid treated tea plants can be caused by the requirement of JA accumulation, which may be a result of hypersensitive reaction to the imidacloprid treatment. In contrast to that of *CsLOX*3, the expression level of *CsHPL* was much lower in the imidacloprid treated tea plants than that in the control plants. The lower expression level of the *CsHPL* gene was consistent with the lower release amount of hexenal in the imidacloprid treated tea plants.

Chemical pesticides are frequently employed and very effective for pest control. Many studies have displayed the phenotypic characters of pests and the natural enemies after pesticide treatment^37–40^. As to the effect of pesticides on treated-plants, insecticide are known to induce changes in plant growth, photosynthesis, reactive-oxygen species metabolism, nodule formation, and N, P, K accumulation^41–43^. To the best of our knowledge, this is the first study on the effects of chemical insecticide on GLVs anabolism and release. Our results demonstrated that the treatment of imidacloprid at the recommended concentration resulted in the suppression of *CsHPL*, and consequently led to the lower release amount of GLVs in tea plants. This suggests that the concentration of imidacloprid should be reassessed to effectively control pests without damaging plant’s indirect defense.

Here, our study merely provides an introduction to the field of research. To clarify the biological mechanisms of pesticides on GLVs anabolism and release, more research is needed to do. Of course, use just imidacloprid treatment is not enough, other kind of pesticides should also be employed. A combination of transcriptomic and metabolomics approaches will be helpful to examine the effects of pesticide at physiological, cytological and molecular levels in plant. To do the studies will help human to get new ideas about balancing the relationship between pest chemical control and plant defensive capacity, which will finally reach the goal of good pest control with less chemical pesticides.

## Methods

### Tea plants

For the pots experiment, cuttings of 1-year-old cloned tea plants (*Camellia sinensis* cv. Longjing 43) were planted in plastic pots individually under controlled conditions in the tea plantation at Anhui Agricultural University, for one year before being used. The healthy and uniform tea plants were selected for the experiments. For the field assay, the same cultivars of 10-year-old tea plants cultivated in the horticultural research station of Anhui Agricultural University were used.

### Chemical pesticide treatment

The selected tea plants were randomly divided into two groups before treatment. One group of tea plants were sprayed evenly with imidacloprid solution at the manufacturer’s (Jiangsu Jianshen Biology Agrochemical Co., Ltd) recommended concentration until the droplets were coalesced and began to drip off the leaves. The other group were sprayed with water as controls. Sprays were applied between 17:30 and 18:00. For each group of treatment, three independent biological replicates were conducted in this study.

### Volatiles collection

Volatile compounds were collected by the dynamic headspace techniques according to the method of Raguso and Pellmyr^44^. The volatiles were trapped in a glass tube which contained 20 mg of 80/100 mesh Porapak Q adsorbent (Sigma, USA). For the pots and field experiments, volatiles collection was done on the third day and the second day after the treatment, respectively. Before the volatiles collection, two same size mechanical injury wounds were sheared on edges of the first and the second fully expanded leaves respectively with autoclaved scissors avoiding of damage to the leaf vein. The collection began immediately after wounding and lasted for 30 min in triplicates. After each collection, the trapped volatiles were eluted using 300 μl methylene dichloride, and then 400 ng of tetrahydronaphthalene was added as an internal standard. Fresh weights of the tested tea leaves was separately weighed after the volatile collection.

### Volatile analysis

Volatile analyses were conducted using a gas chromatograph-mass spectrometer (GC-MS) QP2010 (Shimadzu, Japan) coupled with a DB-5 MS capillary column (J&W Scientific, 30 m × 0.25 mm i.d., 0.25 μm film thickness). The GC-MS equipment has an automated injection system, and was operated in a splitless mode using helium as the carrier gas at a constant flow of 1 ml min^−1^. The injector temperature was 230 °C, the ionization energy was 70 eV, the scanned mass range was from 40 to 600 amu, and the scan frequency was 0.2 sec per scan. After injection, the column temperature was kept for 3 min at 40 °C, then ramped at 10 °C/min to 180 °C, and ramped at 40 °C/min to 220 °C, and held constant for 2 min. Data collection was done by Shimadzu GC ChemStation software. The four C6 volatile components were identified according to the recorded mass spectra compared with both the authentic reference chemicals and the NIST and Wiley spectral databases. Amounts of the volatile compounds were calculated according to the percentages of peak areas relative to that of the internal standard.

### RNA isolation and qRT-PCR analysis

The first and the second fully expanded leaves of tea plants cultivated in the pots were separately collected before and on the third day after imidacloprid treatment. Three independent biological samples were collected at each time points, and the total RNA was isolated according to the modified CTAB method^45^. cDNA was synthesized from the total RNA by employing the Prime Script™ RT reagent Kit (TaKaRa, Dalian, China) following the manufacturer’s instructions. The expression analysis was done with the procedure as follow: 95 °C for 30 s, then 95 °C for 5 s and 60 °C for 30 s by 40 cycles. The reaction was done in 20 μL total volume containing 0.4 μL (10 mM) of each gene-specific primer, 10 μL of 2× SYBR premix *ExTaq*, 2 μL of diluted cDNA as the template, and 7.2 μL of ddH_2_O. The glyceraldehyde-3-phosphate dehydrogenase (*CsGAPDH*) gene of tea plants was used as the internal reference gene. Specific primers for qRT-PCR are presented in Table S1. *CsLOX3* and *CsHPL* gene expression levels were calculated using the 2^−∆∆CT^ method^3^.

## Electronic supplementary material

Table S1
