# Supplementary material for: Effects of Chemical Insecticide Imidacloprid on the Release of C6 Green Leaf Volatiles in Tea Plants (Camellia sinensis)
Source: Sci Rep. 2019 Jan 24;9:625. doi: 10.1038/s41598-018-36556-0 (PMC6345918; doi:10.1038/s41598-018-36556-0)
Supplement: Supplementary file 1 — Table S1 [file 41598_2018_36556_MOESM1_ESM.pdf]

Effects of Chemical Insecticide Imidacloprid on the Release of C_6 Green Leaf Volatiles in Tea Plants (*Camellia sinensis*)

Qiying Zhou, Xi Cheng, Shuangshuang Wang, Shengrui Liu, Chaoling Wei*

Supplementary information

Table S1. Primer sequences used for quantitative real-time PCR

Gene	Primer sequence
CsLOX3	Forward: 5'-ATCCAATGTCGGAGTCG-3'<br>Reverse: 5' -GAAAGCCAAGTTCAGTGTCA-3'
CsHPL	Forward: 5'-ATCCCTAACACCGCCATCG-3'<br>Reverse: 5'-CCTTGGAACCAGAAGTAGTC-3'
CsGAPDH	Forward: 5'-TTGGCATCGTTGAGGGTCT-3'<br>Reverse: 5'-CAGTGGGAACACGGA AAG C-3'
